# Elevated FDG uptake in non-tumorous lung regions does not predict immune checkpoint inhibitor–related pneumonitis in lung cancer patients

**DOI:** 10.3389/fonc.2025.1563030

**Published:** 2025-08-20

**Authors:** Friederike Völter, Lukas Wehlte, Blerina Resuli, Julia Walter, Lea Daisenberger, Maria Ingenerf, Maurice Heimer, Matthias Brendel, Gabriel T. Sheikh, Lena M. Unterrainer, Diego Kauffmann-Guerrero, Thomas Pfluger, Lucie Heinzerling, Amanda Tufman

**Affiliations:** ^1^ Department of Nuclear Medicine, LMU University Hospital, Munich, Germany; ^2^ Department of Medicine IV, Endocrinology, Diabetes and Metabolism, LMU University Hospital, Munich, Germany; ^3^ German Center for Lung Research (Deutsches Zentrum für Lungenforschung (DZL)) (Comprehensive Pneumology Center - Munich (CPC-M)), Munich, Germany; ^4^ Department of Medicine V, LMU University Hospital, Munich, Germany; ^5^ Bayerisches Zentrum für Krebsforschung (BZKF), Munich, Germany; ^6^ Department of Dermatology and Allergy, LMU University Hospital, Munich, Germany; ^7^ Department of Radiology, LMU University Hospital, Munich, Germany; ^8^ Munich Cluster for Systems Neurology (SyNergy), Munich, Germany; ^9^ German Center for Neurodegenerative Diseases (DZNE), Munich, Germany; ^10^ Ahmanson Translational Theranostics Division, Department of Molecular and Medical Pharmacology, David Geffen School of Medicine, University of California, Los Angeles (UCLA), Los Angeles, CA, United States; ^11^ Department of Dermatology, University Hospital Erlangen, Comprehensive Cancer Center Erlangen – European Metropolitan Region Nürnberg, Comprehensive Cancer Center (CCC) Alliance Würzburg, Erlangen, Regensburg, and Augsburg (WERA), Erlangen, Germany

**Keywords:** lung cancer, NSCLC, SCLC, [^18^F]FDG-PET/CT, standardized uptake value, checkpoint inhibitor therapy, checkpoint inhibitor-related pneumonitis, immune-related adverse events

## Abstract

**Background:**

Predictors for checkpoint inhibitor-related pneumonitis (cinrPneumonitis) are desperately needed. This study aimed to investigate the pretreatment standardized uptake value (SUV) on [^18^F]FDG-PET/CT of non-tumorous lung tissue as a predictive imaging marker for the development of cinrPneumonitis in 239 patients with lung cancer.

**Methods:**

All patients with lung cancer receiving [^18^F]Fluorodeoxyglucose positron emission tomography/computed tomography (FDG-PET/CT) prior to immune checkpoint inhibitor (ICI) therapy were included and retrospectively analyzed. Pretreatment SUV_MEAN_, SUV_MAX_, SUV_95_, SUV normalized by lean body mass (SUL_MEAN_, SUL_MAX_) and clinical variables were compared for patients with and without cinrPneumonitis. Logistic regression analyses were performed to identify the predictive value of pretreatment SUV for the development of cinrPneumonitis.

**Results:**

A total of 239 patients were included, of whom 41 (17.2%) developed cinrPneumonitis. The pretreatment radioligand uptake (SUV_MEAN_, SUV_MAX_, SUV_95,_ SUL_MEAN_ and SUL_MAX_) was not significantly elevated in patients who developed cinrPneumonitis. Logistic regression using sex, age, body mass index and chronic obstructive pulmonary disease as covariables additionally showed no significant association between pretreatment radioligand uptake and the risk of cinrPneumonitis. However, an increased likelihood of developing cinrPneumonitis (relative risk = 1.979; *p* = 0.027) was shown in patients who received thoracic radiation during ICI therapy.

**Conclusion:**

This is the largest study on the association of pretreatment radioligand uptake of the non-tumorous lung and the risk of a cinrPneumonitis. Our results showed no significant association between elevated pretreatment radioligand uptake of non-tumorous lung tissue on FDG-PET/CT and the development of cinrPneumonitis.

## 1 Introduction

Lung cancer remains one of the most prevalent and lethal malignancies globally (1, 2). The advent of immune checkpoint inhibitors (ICI) targeting programmed death-1 (anti-PD-1), programmed death ligand-1, and cytotoxic T-lymphocyte antigen-4 (CTLA-4) has significantly improved outcomes in patients with non-small cell lung cancer (NSCLC), demonstrating superior progression-free survival and overall survival rates compared to chemotherapy (3–9). Despite these advancements, the clinical benefits of ICI are frequently compromised by the high incidence of immune-related adverse events (irAEs), which occur in up to 65% of the patients undergoing treatment and can be fatal (10–12). Among these, immune checkpoint inhibitor-related pneumonitis (cinrPneumonitis) is particularly concerning due to its significant prevalence and its potential to impair lung function, thereby adversely impacting overall survival and quality of life (7, 13–18). Furthermore, cinrPneumonitis accounts for 35% of ICI-induced fatalities (19, 20). Severe cases of cinrPneumonitis can necessitate the discontinuation of effective therapies, which further limits ICI benefit (16, 17, 21). Given these challenges, there is a critical need for early identification of patients at heightened risk for cinrPneumonitis. Various factors including chronic obstructive pulmonary disease (COPD) (22), interstitial lung disease (23), tumor histologic type (24), combination therapy with Nivolumab plus Ipilimumab (13), pretreatment radiotherapy (16), and body mass index (BMI) (25) have been evaluated for their predictive value in the development of irAEs and specifically, cinrPneumonitis. Furthermore, pretreatment standardized uptake value (SUV) of non-tumorous lung tissue measured via [^18^F]Fluorodeoxyglucose positron emission tomography/computed tomography (FDG-PET/CT) has been proposed as an imaging biomarker to predict the risk of developing cinrPneumonitis (26–28). The underlying hypothesis is that a pretherapeutically increased metabolic activity in non-tumorous lung tissue reflects a subclinical, low-grade inflammation, which may predispose the tissue to subsequent immune-related pneumonitis through immune priming. However, while SUV on FDG-PET/CT during immunotherapy has proven effective in the early diagnosis of various irAEs including cinrPneumonitis, colitis and thyroiditis in small sample size studies (26–28), a correlation between pretreatment SUV and the subsequent development of therapy-related pneumonitis remains unsubstantiated (26, 27). Our preliminary study indicated that SUV is positively correlated with BMI and negatively associated with COPD (29), making both significant factors to consider when predicting cinrPneumonitis risk using pretreatment SUV.

This study aims to further evaluate the predictive value of pretreatment SUV in non-tumorous lung tissue as a biomarker for the occurrence of cinrPneumonitis in a large cohort of patients undergoing ICI therapy, incorporating BMI and COPD as covariates in our logistic regression model.

## 2 Materials and methods

### 2.1 Patient population

All patients with lung cancer who were treated with ICI at LMU Klinikum between 2014 and 2022 and who received an FDG-PET/CT as part of their staging before the start of immunotherapy were included. Patients who received their FDG-PET/CT more than one year before were excluded. This study was conducted in accordance with the Declaration of Helsinki and approved by the ethics committee of LMU Munich (Project No. 474–16 UE).

### 2.2 Image acquisition

All patients had to fast for at least 4–6 h prior to the injection of [^18^F]FDG and showed a median blood glucose level of 101 mg/dL (range 70–184 mg/dL). A median activity of 240 MBq (range 142–330 MBq) [^18^F]FDG was injected intravenously (3 MBq/kg body weight). If no contraindication was given, furosemide (10–20 mg) and buscopan (20 mg) were administered for a faster excretion and a decreased gastrointestinal radioligand activity. Before PET imaging, a diagnostic CT scan after the administration of 350 mg of iomeprol (Imeron^®^) at 1.5 ml/kg body weight (portal-venous phase) was acquired in mid-breath-hold. PET acquisition was started at a median of 61 min post injection (1^st^ Quartile: 51 min; 3^rd^ Quartile: 73 min) with 2.5 min per bed position. PET images were reconstructed as described previously (30). The SUV was calculated using the following standard calculation (31).

SUV calculation:


$$SUV=\frac{Sphere\;activity\;\left[\frac{Bq}{ml}\right]*body\;weight\;\left(kg\right)\;}{injected\;dose}$$

### 2.3 Image analysis

FDG-PET/CT images were evaluated using Visage^®^7, Visage Imaging, Inc. (San Diego, CA, USA; version 7.1.18). Four 3-dimensional spheres with a diameter of 30 millimeters were placed into lung parenchyma, ensuring placement in regions not infiltrated by lung tumor, carefully excluding areas with large blood vessels, focal consolidations or pleural effusion. One sphere was placed in each of the upper and lower lobe of both the right and left lung, as described in a previous study (29). Spheres were excluded when it was not possible to place them exclusively in non-tumorous lung tissue (e.g., due to tumor infiltration, metastasis, or previous lobectomy). The mean SUV (SUV_MEAN_), maximum SUV (SUV_MAX_), and the standard deviation (SD) of the SUV (SUV_SD_) were measured for each sphere. Mean values of all four spheres were calculated for each patient for the SUV_MEAN_ and SUV_MAX_. Additionally, mean values were computed for the two upper spheres, the two lower spheres and two spheres contralateral to the tumor (tumor free lung, TFL). Additionally, the 95^th^ percentile of SUV_MEAN_ (SUV_95_) was calculated for all four spheres with the following formula:

SUV_95_ calculation:


$$SUV_{95}=SUV_{MEAN}\;+\;\left(qnorm\left(0.95\right)*SUV_{SD}\right)$$

Furthermore, SUV were normalized by lean body mass (SUL_MEAN_, SUL_MAX_) with the Janmahasatian formula to correct for falsely high SUV in obese patients (32).

SUL calculation:


$$LBM_{men}\;=1.10*body\;weight\;\left(kg\right)-128\mathrm{*BMI *}body\;weight\;\left(kg\right)$$

$$LBM_{women}=1.07*body\;weight\;\left(kg\right)-148\mathrm{*BMI*}body\;weight\;\left(kg\right)$$

$$\text{SUL}=\frac{SUV}{body\;weight\;\left(kg\right)*LBM}$$

### 2.4 Diagnosis of cinrPneumonitis

Pneumonitis was diagnosed by the treating pulmonologists based on imaging reports and respiratory symptoms and graded using Common Terminology Criteria for Adverse Events (CTCAE) version 5.0. A semi-automatic tool was built to analyze the text of electronic patient records using keywords for patient characteristics and pneumonitis diagnosis with consideration for possible spelling errors. When keywords were found, the text was reviewed manually. All collected data were anonymized and stored in a Microsoft Excel database (Redmond, WA, USA; version 2108).

### 2.5 Statistical analysis

Patients were divided in groups depending on whether they developed a cinrPneumonitis. Numeric variables (including SUV and SUL) of patients with and without cinrPneumonitis were compared using Mann–Whitney U test or T-test depending on whether they showed normal distribution according to the test of normality by Lilliefors. For all significant variables, the relative risk was calculated. Differences in proportions of categorical variables were analyzed using Pearson’s chi^2^ test or Fisher-Yates-Test depending on group size. Kruskal-Wallis-Test was used to test if PET parameters acquired with different PET/CT devices were significantly different. Multiple logistic regression correcting for sex, age, BMI, COPD and thorax radiation after the pretreatment FDG-PET/CT was used to further investigate a possible association of pretreatment tracer uptake (SUV_MEAN_, SUV_MAX_, SUV_95_, SUL_MEAN_, and SUL_MAX_) with the risk of cinrPneumonitis. All statistical analysis and plots were conducted using R-Studio (Boston, MA, USA; version 2024.04.2 + 764) and R (Vienna, Austria; version 4.4.0). *A priori* power analysis was performed using G*Power (version 3.1.9.6; Düsseldorf, Germany). It was assumed that a change in radioligand uptake would be clinically relevant if SUV_MAX_ or SUV_MEAN_ increased by more than 30% in patients with cinrPneumonitis, compared to those without, adopted from the PERCIST criteria. Additionally, the probability of cinrPneumonitis was estimated at 15%. Based on previous data, the median SUV_MAX_ was approximately 1.0 with a SD of 0.5, and the median SUV_MEAN_ was approximately 0.5 with an SD of 0.2. Under these assumptions, a statistical power of 0.95 would be achieved with 25 patients with cinrPneumonitis and 194 without for SUV_MAX_, and 23 patients with cinrPneumonitis and 157 without for SUV_MEAN_.

## 3 Results

### 3.1 Study population

A total of 293 patients with lung cancer underwent ICI therapy during the observation period. 53 patients were excluded because the FDG-PET/CT was acquired after therapy with immune checkpoint inhibitors was started. In one patient, the placement of spheres in non-tumorous lung tissue was not feasible due to a prior lung operation and extensive tumor infiltration. The baseline characteristics of the remaining 239 patients are presented in **Table 1**.

**Table 1:** Distribution of clinical characteristics in the two cohorts of lung cancer patients who developed or did not develop pneumonitis after therapy with checkpoint inhibitors.

Characteristics	All	No Pneumonitis	Pneumonitis	*p-value*
Patients	n = 239	n = 198 (82.9%)	n = 41 (17.2%)	
Physical characteristics				
Sex				
**Female**	91/239 (38.1%)	78/198 (39.4%)	13/41 (31.7%)	0.456
**Male**	148/239 (61.9%)	120/198 (60.6%)	28/41 (68.3%)	0.456
**Age [y]**	67.6 (± 10.9)	68.2 (± 10.6)	64.5 (± 11.8)	0.065
**BMI [kg/m^2^]**	24.5 (± 4.7)	24.4 (± 4.8)	24.9 (± 4.1)	0.326
Smoking history				
**Nicotine consumption**	197/239 (82.4%)	164/198 (82.8%)	33/41 (80.5%)	0.822
**Packyears [y]**	34.3 (± 27.0)	34.5 (± 26.4)	33.7 (± 29.8)	0.558
Blood count				
**Hb [g/dL]**	11.9 (± 2.3)	11.9 (± 2.2)	12.0 (± 2.6)	0.831
Tumor				
Staging				
**I**	2/136 (1.5%)	2/114 (1.8%)	0/22 (0.0%)	1.000
**II**	46/136 (33.8%)	38/114 (33.3%)	8/22 (36.4%)	0.808
**III**	87/136 (64%)	73/114 (64%)	14/22 (63.6%)	1.000
**IV**	1/136 (0.7%)	1/114 (0.9%)	0/22 (0%)	1.000
Histology				
**NSCLC adenocarcinoma**	136/239 (56.9%)	113/198 (57.1%)	23/41 (56.1%)	1.000
**NSCLC squamous cell carcinoma**	65/239 (27.2%)	54/198 (27.3%)	11/41 (26.8%)	1.000
**SCLC neuroendocrine carcinoma**	24/239 (10.0%)	21/198 (10.6%)	3/41 (7.3%)	0.775
**Other lung cancer histology^1^**	14/239 (5.9%)	10/198 (5.1%)	4/41 (9.8%)	0.269
Clinical history				
**Lung operation**	20/239 (8.4%)	15/198 (7.6%)	5/41 (12.2%)	0.352
**Thorax radiation**	125/239 (52.3%)	96/198 (48.5%)	29/41 (70.7%)	0.015
**- Thorax radiation before PET/CT**	62/239 (25.9%)	50/198 (25.3%)	12/41 (29.3%)	0.735
**- Thorax radiation after PET/CT**	63/239 (26.4%)	46/198 (23.2%)	17/41 (41.5%)	0.027
**Combination with Chemotherapy**	163/239 (68.2%)	132/198 (66.7%)	31/41 (75.6%)	0.357
Immune checkpoint inhibitors				
**Pembrolizumab**	130/239 (54.4%)	111/198 (56.1%)	19/41 (46.3%)	0.335
**Nivolumab**	73/239 (30.5%)	55/198 (27.8%)	18/41 (43.9%)	0.064
**Atezolizumab**	31/239 (13.0%)	28/198 (14.1%)	3/41 (7.3%)	0.312
**Nivolumab and Ipilimumab**	9/239 (3.8%)	6/198 (3.0%)	3/41 (7.3%)	0.187
**Other Immune checkpoint inhibitors^2^**	16/239 (6.7%)	15/198 (7.6%)	1/41 (2.4%)	0.320
**Subsequent therapy with ≥2 ICIs^3^**	19/239 (7.9%)	16/198 (8.1%)	3/41 (7.3%)	1.000
Comorbidities				
**COPD**	57/239 (23.8%)	48/198 (24.2%)	9/41 (22.0%)	0.842
**Bronchial asthma**	7/239 (2.9%)	5/198 (2.5%)	2/41 (4.9%)	0.344
**Pericardial effusion**	14/239 (5.9%)	9/198 (4.5%)	5/41 (12.2%)	0.070
**Pleural effusion**	56/239 (23.4%)	45/198 (22.7%)	11/41 (26.8%)	0.717
**Type II diabetes**	35/238 (14.7%)	33/197 (16.8%)	2/41 (4.9%)	0.054
**Coronary heart disease**	34/239 (14.2%)	27/198 (13.6%)	7/41 (17.1%)	0.623
PET/CT device				
**Siemens Biograph 20**	42/239 (17.6%)	38/198 (19.2%)	4/41 (9.8%)	0.180
**Siemens Biograph 40**	19/239 (7.9%)	15/198 (7.6%)	4/41 (9.8%)	0.750
**Siemens Biograph 64**	14/239 (5.9%)	12/198 (6.1%)	2/41 (4.9%)	1.000
**GE Discovery 690**	136/239 (56.9%)	111/198 (56.1%)	25/41 (61%)	0.685
**Philips Guardian Body**	12/239 (5.0%)	11/198 (5.6%)	1/41 (2.4%)	0.697
**Other PET/CT devices^4^**	16/239 (6.7%)	11/198 (5.6%)	5/41 (12.2%)	0.161

Comparison of proportions in patients with pneumonitis and patients without pneumonitis using Pearson’s chi2 test or Fisher-Yates-Test, numerical variables were compared with Mann–Whitney U test or T-test.

^1^Other lung cancer histology: poorly differentiated lung carcinoma (n = 4), adenosquamous lung carcinoma (n = 3), NSCLC neuroendocrine tumor (n = 3), mucoepidermoid carcinoma (n = 2), pleomorphic carcinoma (n = 1), sarcomatoid carcinoma (n = 1).

^2^Other immune checkpoint inhibitors: Durvalumab (n = 7), Dostarlimab (n = 1), single Ipilimumab (n = 1).

^3^Subsequent therapy with ≥2 ICIs: more than one immune checkpoint-inhibitor was given before the occurrence of cinrPneumonitis (patients with a combination therapy of nivolumab and ipilimumab are listed separately).

^4^Other scanners: Siemens Biograph 128 (n = 2), Siemens 1094 (n = 4), Philips Medical Systems GEMINI TF Big Bore (n = 2), Philips Vereos (n = 3), Philips Medical Systems GEMINI TF TO F16 (n = 3), GE MEDICAL SYSTEMS Discovery 600 (n = 1), CPS 1024 (n = 1).

BMI, Body mass index; NSCLC, non-small cell lung cancer; SCLC, small cell lung cancer; ICI, immune checkpoint inhibitors; COPD, chronic obstructive pulmonary disease; PET/CT, Positron emission tomography/computed tomography.

### 3.2 Evaluation of cinrPneumonitis

Following immunotherapy, 41 patients (17.2%) developed cinrPneumonitis. According to CTCAE, there were 10 patients (24.4%) with grade 1 pneumonitis, 16 patients (39.0%) with grade 2 pneumonitis, 9 patients (22.0%) with grade 3 pneumonitis, 3 patients (7.3%) with grade 4 pneumonitis, and 3 patients (7.3%) who died from respiratory compromise (grade 5). The median interval between the start of ICI therapy and cinrPneumonitis was 130 days (1QU = 58 days; 3QU = 211 days). In the cohort of patients who developed pneumonitis, a significantly greater proportion underwent concurrent thoracic radiation in addition to ICI therapy compared to those without pneumonitis (41.5% vs. 23.2%; *p* = 0.027; relative risk = 1.979). The median time between irradiation and the development of a pneumonitis was 189 days (1QU = 153 days; 3QU = 318 days). Patients with lung radiation before the start of ICI therapy did not show an increased risk of pneumonitis compared to patients without lung radiation before ICI therapy. The median time between the lung radiation before the FDG-PET/CT and the beginning of ICI therapy was 202.5 days (1QU = 92.25 days; 3QU = 481 days). Patients with preexisting type II diabetes showed a trend toward a lower risk of developing cinrPneumonitis compared to patients without diabetes (relative risk: 0.297; *p* = 0.054).

### 3.3 Comparability of radioligand uptake measured on different PET/CT devices

The comparability of SUV and SUL obtained from different PET/CT devices was tested with Kruskal-Wallis-Test (**Supplementary Table S1**). While there was no significant difference between SUV_MEAN_ values (*p* = 0.193 – 0.356), SUV_95_ (*p* = 0.074 – 0.591), and SUL_MEAN_ (*p* = 0.263 – 0.433) across all devices, there were significant differences of SUV_MAX_ of the whole lung, lower lung and the tumor free lung (*p* = 0.007; *p* = 0.030), and SUL_MAX_ of the tumor free lung (*p* = 0.048). For further analyses with SUV_MAX_ and SUL_MAX_, the subgroup of 136 patients scanned with the same PET/CT device was used.

### 3.4 Comparison between pretreatment tracer uptake and occurrence of pneumonitis

**Figure 1:**
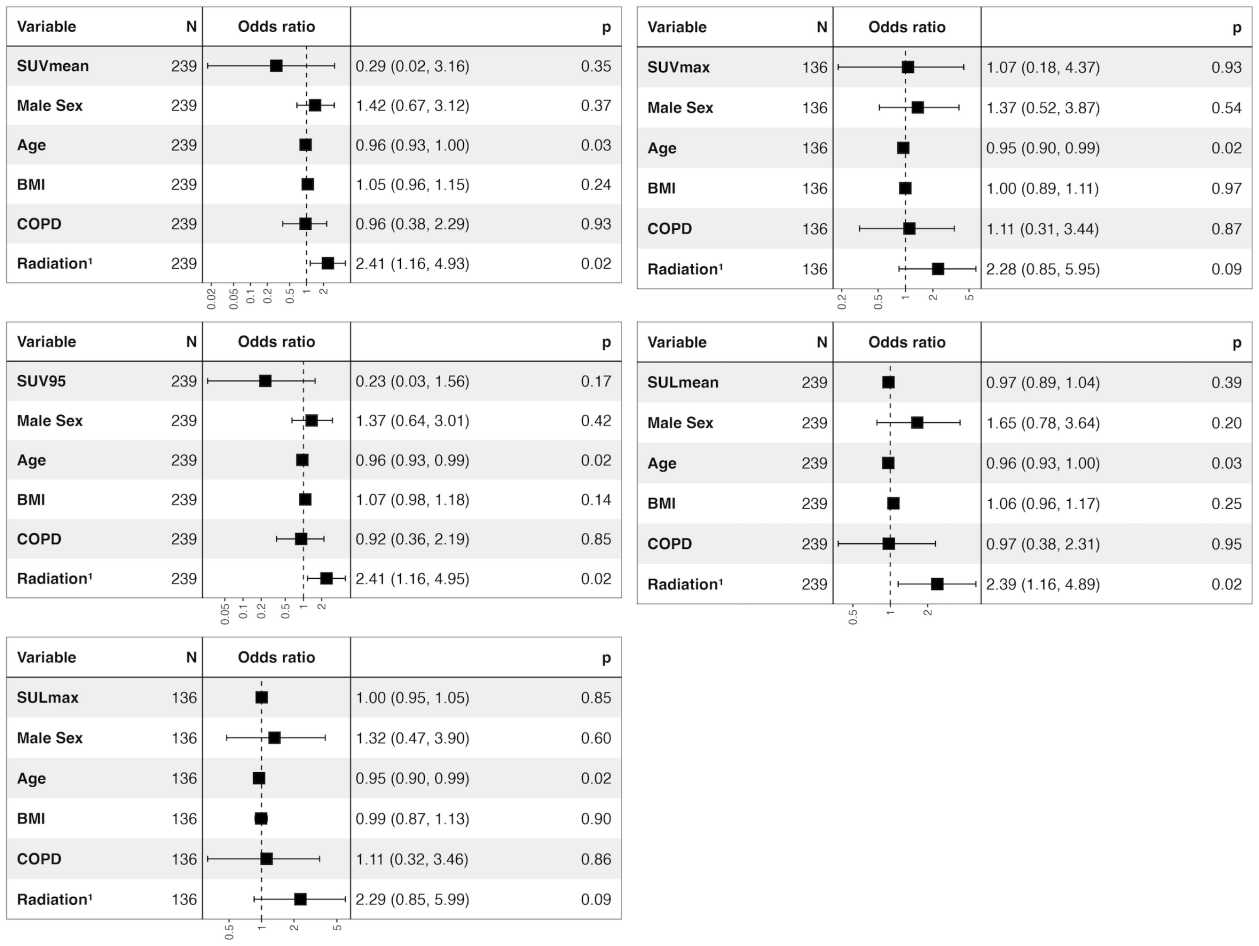
Forest plot of logistic regression with pneumonitis as response and SUV_MEAN_, SUV_MAX_, SUV_95_, SUL_MEAN_, and SUL_MAX_ as independent variables. Odds Ratio (95% confidence interval). ^1^Radiation therapy during ICI therapy. SUV, standardized uptake value; SUL, standardized uptake value normalized by lean body mass. COPD, Chronic obstructive pulmonary disease; BMI, Body mass index.

Patients who developed cinrPneumonitis after the initiation of ICI therapy did not show increased pretreatment radioligand uptake of the whole lung, the upper lung, the lower lung or the tumor free lung (*p* = 0.457 – 0.971). Results are displayed in detail in **Table 2**, **Supplementary Tables S2, S3** and in **Supplementary Figure S1**. Additionally, the logistic regression analysis with cinrPneumonitis as response and SUV or SUL as independent variables using sex, age, BMI, COPD and thorax radiation after the pretreatment FDG-PET/CT as covariables did not show a significant association of tracer uptake and the risk of cinrPneumonitis (**Figure 1**). However, the patient’s age was significantly associated with the risk of cinrPneumonitis (β = -0.054 – -0.037; *p* = 0.020 – 0.028). Additionally, thorax radiation during ICI therapy was significantly associated with the risk of cinrPneumonitis when using SUV_MEAN_, SUL_MEAN_ or SUV_95_ as independent variables (β = 0.872 – 0.880; *p* = 0.016 – 0.017).

**Table 2:** SUV variables compared with the occurrence of pneumonitis during immunotherapy.

Mean; SD	No Pneumonitis		Pneumonitis		*p* value
	n = 198 (82.8%)		n = 41 (17.2%)		
SUV_MEAN_					
whole lung	0.48	(± 0.19)	0.46	(± 0.18)	0.678
upper lung	0.44	(± 0.19)	0.44	(± 0.19)	0.971
lower lung	0.51	(± 0.22)	0.49	(± 0.19)	0.686
TFL	0.52	(± 0.24)	0.49	(± 0.21)	0.495
SUV_MAX_^1^					
whole lung	0.98	(± 0.35)	0.99	(± 0.42)	0.915
upper lung	0.91	(± 0.34)	0.95	(± 0.43)	0.711
lower lung	1.05	(± 0.44)	1.03	(± 0.43)	0.805
TFL	1.07	(± 0.49)	1.02	(± 0.37)	0.560
SUV_95_					
whole lung	0.67	(± 0.26)	0.64	(± 0.25)	0.626
upper lung	0.62	(± 0.25)	0.57	(± 0.18)	0.542
lower lung	0.72	(± 0.34)	0.65	(± 0.21)	0.479
TFL	0.74	(± 0.45)	0.67	(± 0.25)	0.465
SUL_MEAN_					
whole lung	14.49	(± 6.88)	14.58	(± 6.52)	0.757
upper lung	13.46	(± 6.87)	13.74	(± 6.49)	0.457
lower lung	15.51	(± 7.99)	15.41	(± 7.01)	0.904
TFL	15.75	(± 8.64)	15.40	(± 7.75)	0.920
SUL _MAX_^1^					
whole lung	30.12	(± 13.91)	30.59	(± 14.89)	0.960
upper lung	28.00	(± 13.02)	29.16	(± 14.75)	0.720
lower lung	32.23	(± 16.51)	32.03	(± 15.70)	0.964
TFL	33.17	(± 18.54)	31.80	(± 14.80)	0.871

^1^For this calculation, the subgroup of 136 patients scanned with the same PET device was used (25/41 of all patients with pneumonitis, 111/198 patients without pneumonitis). SUV, standardized uptake value; SUL, standardized uptake value normalized by lean body mass; TFL, contralateral lung compared to the side of the tumor.

## 4 Discussion

This study, to our knowledge, is the largest to date investigating the association between pretreatment radioligand uptake in non-tumorous lung and the risk of cinrPneumonitis (26, 27, 33, 34). Our findings demonstrate no significant difference in radioligand uptake between patients who developed cinrPneumonitis and those who did not, even after controlling for covariables such as sex, age, BMI and COPD in a logistic regression model. Thoracic radiation therapy during ICI treatment was significantly associated with an elevated risk of cinrPneumonitis, whereas prior radiation therapy did not increase the risk. The observed incidence of cinrPneumonitis in our cohort (17.2%) aligns with reported rates in the literature ranging between 9.5% and 21.8% (15, 17, 35–38).

Identifying predictive biomarkers for the development of cinrPneumonitis is crucial given its status as the most lethal adverse event of ICI therapy. Early detection of patients at risk prior to therapy initiation could inform preventive strategies, such as stringent monitoring or alternative treatment approaches (39). Furthermore, evidence from other irAEs, such as ICI-induced myocarditis underscores the importance of early intervention in mitigating adverse outcomes (39, 40).

FDG-PET has proven useful for diagnosing irAEs at an early stage, with small-scale studies reporting elevated radioligand uptake in non-target organs during irAEs, including pneumonitis, thyroiditis and colitis (26–28): A visually increased regional tracer uptake has been observed during irAEs across a range of organs, including colitis, gastritis, thyroiditis, myositis/arthritis, hepatitis, pneumonitis, sarcoid-like reaction, and hypophysitis in a small sample size study with 46 patients (27). Another small study with 18 participants focusing on checkpoint-inhibitor associated thyroiditis in lung cancer patients found that SUV_MEAN_, SUV_MAX_, and total lesion glycolysis of the thyroid were significantly increased in patients experiencing thyroiditis (n = 6) compared to those without thyroiditis (n = 12) (28) and that FDG-PET showed an enhanced uptake even before laboratory changes of thyroid stimulating hormone became apparent. Another study established thresholds for the SUV_95_ of different non-target organs for a PET-based diagnosis of therapy-associated colitis, pneumonitis, and thyroiditis, evaluating a cohort of 58 patients during anti-PD-1 or anti-CTLA-4 ICI therapy including 18 patients with irAEs (26) - they observed significantly elevated SUV_95_ values in organs affected by therapy-associated inflammation.

While FDG-PET enables an early diagnosis of irAEs, a pre-therapeutic risk-prediction for cinrPneumonitis is not possible yet (41). Our study demonstrated no significant association between pretreatment FDG-PET uptake and cinrPneumonitis risk. The pretreatment radioligand uptake was nearly identical between patients who experienced cinrPneumonitis and those without pulmonary side effects during or after ICI therapy. This finding contrasts with the results of two smaller studies suggesting an association between pretreatment normal-lung SUV and subsequent cinrPneumonitis. One study with a small number of 8 patients experiencing cinrPneumonitis reported a significant association of pre-therapeutic FDG-PET and subsequent cinrPneumonitis using a predictive radiomics score including the density of the lung volume and the radioligand uptake (33). Additionally, a very recent study of September 2024 showed that the pretreatment SUV_MEAN_ of the contralateral lung was more frequently elevated >2.09 in the 28 patients with cinrPneumonitis (46%, n=13/28) compared to the patients without cinrPneumonitis (24,1%, n = 33/137) (34). In the latter study, an automatic segmentation of the contralateral lung was used, excluding regions with an SUV above 2.5 to avoid areas with contralateral lung metastases. However, this method may have inadvertedly included lung metastases in the segmented volume. Especially small lung metastases can have an SUV below 2.5 (42, 43). A recent study on the prognostic value of the pretreatment radioligand uptake of lung cancer lesions reported an SUV_MAX_ below 2.5 in 43.1% of the included 108 patients with NSCLC (44). The presence of lung metastases is a known risk factor for cinrPneumonitis (45, 46): A study from 2020 including 253 patients treated with ICI therapy and 15 cases with cinrPneumonitis (among them 61% with lung cancer) reported an OR of 10.8 for lung regions affected by the primary tumor or metastasis (45). Another study from 2022 including 110 patients treated with Durvalumab showed that lung metastases were significantly associated with the risk of cinrPneumonitis with an OR of 10.08 (95% CI = 1.69–199.81; p = 0.076) (46). In our study, the volume of interest was meticulously placed in non-tumorous lung regions to exclude areas potentially affected by metastases, larger blood vessels or consolidations. Consequently, among the 239 patients included in our study only one patient exhibited an SUV_MEAN_ > 2.09 in the non-tumorous lung. This rigorous methodological approach mitigates the potential confounding effects of including metastatic regions, thereby increasing the reliability of our findings.

Furthermore, in our cohort, thoracic radiation therapy during ICI treatment was significantly associated with an elevated risk of cinrPneumonitis. This elevated risk has been shown in multiple studies evaluating therapy-related pneumonitis in patients (47, 48) and in mice (49) with lung cancer. While radiation therapy prior to systemic therapy including ICI therapy and chemotherapy has been identified as a risk factor for radiation-recall pneumonitis (15, 50), our study did not find an increased risk for cinrPneumonitis with prior radiation therapy. Despite the elevated risk of adverse events, combined systemic and radiation therapy remains a standard of care in locally advanced disease (51–53).

In our patient cohort, patients with cinrPneumonitis were younger than patients without cinrPneumonitis, although statistical significance was not reached. This observation is supported by a large US FDA analysis of 51,166 cinrPneumonitis, which found a higher incidence of cinrPneumonitis in patients younger than 60 years compared to patients older than 60 years (54). Additionally, we observed a non-significant trend suggesting a decreased risk of developing cinrPneumonitis in patients with type II diabetes. Previous studies have identified diabetes as a predictive factor for radiation pneumonitis, other studies investigating the association between diabetes and cinrPneumonitis have not found a significant association (17, 55–57).

We found no significant associations between cinrPneumonitis and comorbidities such as COPD, asthma, smoking or an increased BMI. While some studies reported similar findings (16, 38, 58), others have identified lung emphysema, interstitial lung disease, current smoking and an increased BMI as risk factors (15–17, 40, 55, 59). Deng et al. demonstrated the importance of distinguishing between current and former smokers, finding that while former smokers did not have an elevated risk for cinrPneumonitis, current smoking significantly increased the risk (15).

Despite extensive research efforts, the search for predictive markers for ICI-induced toxicity has yet to yield clinically applicable results (60, 61). This study highlights the complexity of factors potentially contributing to cinrPneumonitis and demonstrates that elevated tracer uptake on pre-therapeutic FDG-PET/CT does not correlate with an increased risk of developing cinrPneumonitis.

Certain limitations need to be considered when interpreting this study. First, examinations from multiple PET scanners have been used for this analysis. Optimal comparability of radioligand uptake ideally requires using the same PET scanner. Different reconstruction and post-processing methods affect foremost SUV_MAX_ measurements. To address potential confounders, we conducted all statistical tests on the entire patient dataset and a subgroup scanned with the most frequently used PET/CT scanner, finding no predictive value in pretreatment SUV for cinrPneumonitis. Additionally, the retrospective design of this study imposes a limitation. Since the medical documentation was not originally intended for this research, clinical information about patients’ comorbidities may be incomplete.

## 5 Conclusion

While FDG-PET has been shown to enable an early detection of immune-related adverse events, our findings indicate that pretreatment SUV and SUL values in non-tumorous lung tissue are not predictive for cinrPneumonitis. Consequently, lung cancer patients with a pretreatment elevated tracer uptake in non-tumorous lung regions do not have a higher risk for cinrPneumonitis compared to those without elevated pretreatment uptake values.

## Data Availability

The datasets presented in this study can be found in online repositories. The names of the repository/repositories and accession number(s) can be found in the article/**Supplementary Material**.
