# Supplementary material for: Elevated FDG uptake in non-tumorous lung regions does not predict immune checkpoint inhibitor–related pneumonitis in lung cancer patients
Source: Front Oncol. 2025 Aug 20;15:1563030. doi: 10.3389/fonc.2025.1563030 (PMC12405330; doi:10.3389/fonc.2025.1563030)
Supplement: Supplementary file 1 [file DataSheet1.docx]

Supplementary Material

## Supplementary Figures

**
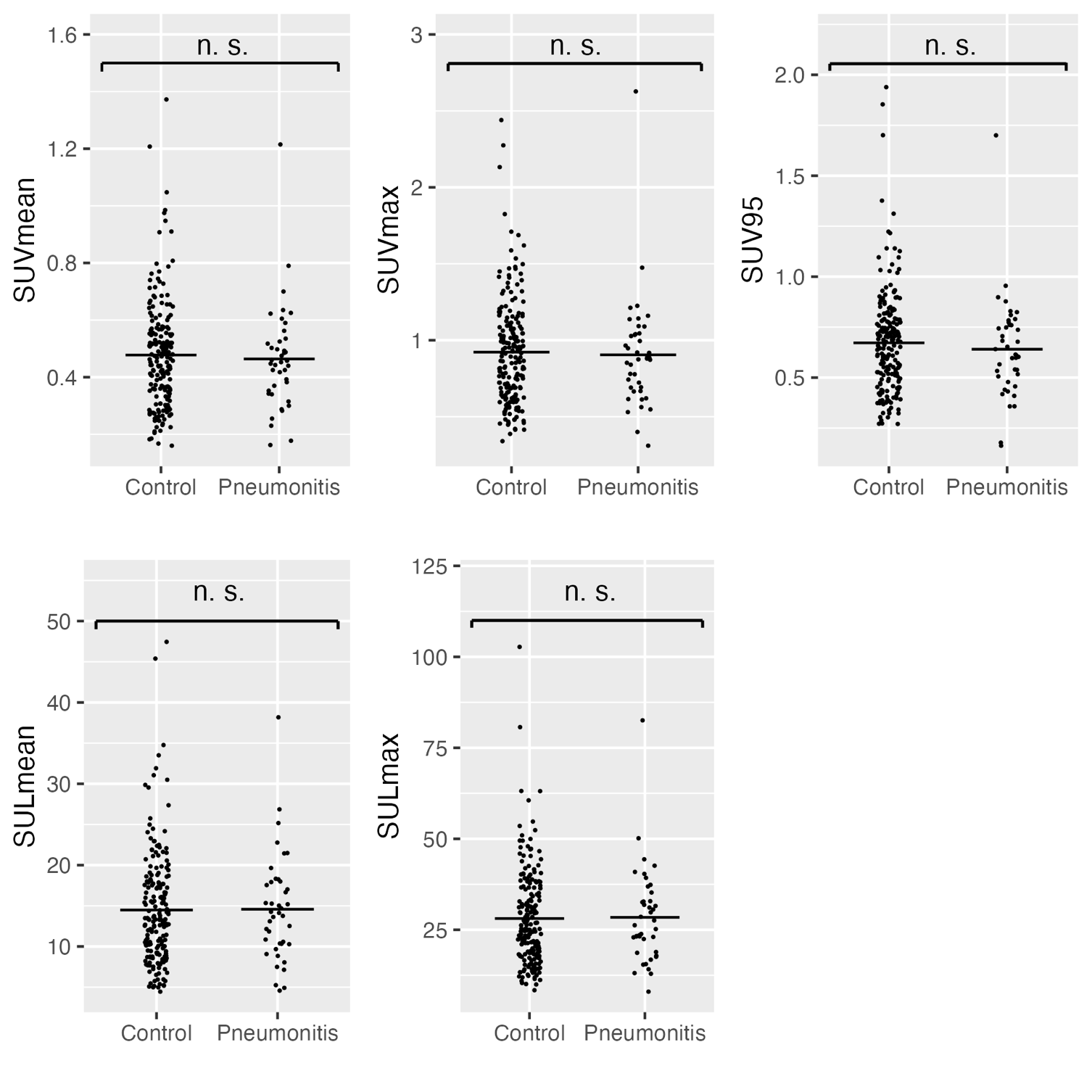
**

**Supplementary Figure 1.** Overview of SUV_MEAN_, SUV_MAX_, SUV_95_, SUL_MEAN_ and SUL_MAX_ in patients with and without immune checkpoint inhibitor-related pneumonitis. SUV = standardized uptake value, SUL = standardized uptake value normalized by lean body mass.
