# Supplementary material for: Elevated FDG uptake in non-tumorous lung regions does not predict immune checkpoint inhibitor–related pneumonitis in lung cancer patients
Source: Front Oncol. 2025 Aug 20;15:1563030. doi: 10.3389/fonc.2025.1563030 (PMC12405330; doi:10.3389/fonc.2025.1563030)
Supplement: Supplementary file 2 [file Table1.docx]

Supplementary Material

**Supplementary Table 1**. Comparison of SUV measurements across different PET-CT scanners (n = 12).

| SUV Measurements | *p* value |  |
| --- | --- | --- |
| SUV_MEAN_ |  |  |
| whole lung | 0.356 |  |
| upper lung | 0.203 |  |
| lower lung | 0.246 |  |
| TFL | 0.193 |  |
| SUV_MAX_ |  |  |
| whole lung | 0.030 | * |
| upper lung | 0.160 |  |
| lower lung | 0.011 | * |
| TFL | 0.007 | ** |
| SUV95 |  |  |
| whole lung | 0.366 |  |
| upper lung | 0.591 |  |
| lower lung | 0.104 |  |
| TFL | 0.074 |  |
| SUL_MEAN_ |  |  |
| whole lung | 0.433 |  |
| upper lung | 0.411 |  |
| lower lung | 0.263 |  |
| TFL | 0.295 |  |
| SUL_MAX_ |  |  |
| whole lung | 0.131 |  |
| upper lung | 0.370 |  |
| lower lung | 0.050 |  |
| TFL | 0.048 | * |
| * = p-value < 0.05, ** = p-value < 0.01. Adjusted alpha after Bonferroni correction: p-value < 0.0025, SUV = standardized uptake value, SUL = standardized uptake value normalized by lean body mass, TFL = two left or right spheres depending on tumor side. | | |
