# Supplementary material for: Elevated FDG uptake in non-tumorous lung regions does not predict immune checkpoint inhibitor–related pneumonitis in lung cancer patients
Source: Front Oncol. 2025 Aug 20;15:1563030. doi: 10.3389/fonc.2025.1563030 (PMC12405330; doi:10.3389/fonc.2025.1563030)
Supplement: Supplementary file 3 [file Table2.docx]

Supplementary Material

**Supplementary Table 2.** SUV variables compared with the occurrence of pneumonitis during immunotherapy of all patients.

|  | | No Pneumonitis | | | | Pneumonitis | | | | *p* value |
| --- | --- | --- | --- | --- | --- | --- | --- | --- | --- | --- |
|  | | n = 198 (82.8%) | | | | n = 41 (17.2%) | | | |  |
| SUV_MEAN_ |  | | |  | | |  | |  |  |
| whole lung | 0.48 | | (±0.19) | | 0.46 | | | (±0.18) | | 0.678 |
| upper lung | 0.44 | | (±0.19) | | 0.44 | | | (±0.19) | | 0.971 |
| lower lung | 0.51 | | (±0.22) | | 0.49 | | | (±0.19) | | 0.686 |
| TFL | 0.52 | | (±0.24) | | 0.49 | | | (±0.21) | | 0.495 |
| SUV_MAX_ |  | |  | |  | | |  | |  |
| whole lung | 0.92 | | (±0.35) | | 0.90 | | | (±0.37) | | 0.764 |
| upper lung | 0.88 | | (±0.35) | | 0.86 | | | (±0.37) | | 0.825 |
| lower lung | 0.97 | | (±0.40) | | 0.94 | | | (±0.39) | | 0.898 |
| TFL | 0.99 | | (±0.45) | | 0.93 | | | (±0.34) | | 0.794 |
| SUV_95_ |  | |  | |  | | |  | |  |
| whole lung | 0.67 | | (±0.26) | | 0.64 | | | (±0.25) | | 0.626 |
| upper lung | 0.62 | | (±0.25) | | 0.57 | | | (±0.18) | | 0.542 |
| lower lung | 0.72 | | (±0.34) | | 0.65 | | | (±0.21) | | 0.479 |
| TFL | 0.74 | | (±0.45) | | 0.67 | | | (±0.25) | | 0.465 |
| SUL_MEAN_ |  | |  | |  | | |  | |  |
| whole lung | 14.49 | | (±6.88) | | 14.58 | | | (±6.52) | | 0.757 |
| upper lung | 13.46 | | (±6.87) | | 13.74 | | | (±6.49) | | 0.457 |
| lower lung | 15.51 | | (±7.99) | | 15.41 | | | (±7.01) | | 0.904 |
| TFL | 15.75 | | (±8.64) | | 15.40 | | | (±7.75) | | 0.920 |
| SUL _MAX_ |  | |  | |  | | |  | |  |
| whole lung | 28.10 | | (±13.27) | | 28.41 | | | (±13.02) | | 0.744 |
| upper lung | 26.74 | | (±13.12) | | 27.08 | | | (±12.70) | | 0.678 |
| lower lung | 29.45 | | (±14.94) | | 29.75 | | | (±14.03) | | 0.746 |
| TFL | 30.10 | | (±16.60) | | 29.44 | | | (±13.09) | | 0.831 |
| SUV = standardized uptake value, SUL = standardized uptake value normalized by lean body mass, TFL = contralateral lung compared to the side of the tumor. | | | | | | | | | | |
