# Supplementary material for: Elevated FDG uptake in non-tumorous lung regions does not predict immune checkpoint inhibitor–related pneumonitis in lung cancer patients
Source: Front Oncol. 2025 Aug 20;15:1563030. doi: 10.3389/fonc.2025.1563030 (PMC12405330; doi:10.3389/fonc.2025.1563030)
Supplement: Supplementary file 4 [file Table3.docx]

Supplementary Material

**Supplementary Table 3.** SUV variables compared with the occurrence of pneumonitis during immunotherapy of subgroup.

|  | | No Pneumonitis | | | | Pneumonitis | | | | *p* value |
| --- | --- | --- | --- | --- | --- | --- | --- | --- | --- | --- |
|  | | n = 111 (81.6%) | | | | n = 25 (18.4%) | | | |  |
| SUV_MEAN_ |  | | |  | | |  | |  |  |
| whole lung | 0.48 | | (±0.19) | | 0.48 | | | (±0.20) | | 0.957 |
| upper lung | 0.42 | | (±0.18) | | 0.45 | | | (±0.22) | | 0.417 |
| lower lung | 0.53 | | (±0.24) | | 0.51 | | | (±0.21) | | 0.542 |
| TFL | 0.55 | | (±0.27) | | 0.51 | | | (±0.24) | | 0.504 |
| SUV_MAX_ |  | |  | |  | | |  | |  |
| whole lung | 0.98 | | (±0.35) | | 0.99 | | | (±0.42) | | 0.915 |
| upper lung | 0.91 | | (±0.34) | | 0.95 | | | (±0.43) | | 0.711 |
| lower lung | 1.05 | | (±0.44) | | 1.03 | | | (±0.43) | | 0.805 |
| TFL | 1.07 | | (±0.49) | | 1.02 | | | (±0.37) | | 0.560 |
| SUV_95_ |  | |  | |  | | |  | |  |
| whole lung | 0.69 | | (±0.27) | | 0.67 | | | (±0.28) | | 0.864 |
| upper lung | 0.61 | | (±0.24) | | 0.58 | | | (±0.19) | | 0.540 |
| lower lung | 0.77 | | (±0.38) | | 0.66 | | | (±0.20) | | 0.354 |
| TFL | 0.80 | | (±0.54) | | 0.70 | | | (±0.27) | | 0.512 |
| SUL_MEAN_ |  | |  | |  | | |  | |  |
| whole lung | 14.61 | | (±7.07) | | 14.79 | | | (±7.19) | | 0.875 |
| upper lung | 12.86 | | (±6.61) | | 13.89 | | | (±7.40) | | 0.438 |
| lower lung | 16.35 | | (±8.64) | | 15.70 | | | (±7.51) | | 0.707 |
| TFL | 16.84 | | (±9.62) | | 15.79 | | | (±8.73) | | 0.613 |
| SUL _MAX_ |  | |  | |  | | |  | |  |
| whole lung | 30.12 | | (±13.91) | | 30.59 | | | (±14.89) | | 0.960 |
| upper lung | 28.00 | | (±13.02) | | 29.16 | | | (±14.75) | | 0.720 |
| lower lung | 32.23 | | (±16.51) | | 32.03 | | | (±15.70) | | 0.964 |
| TFL | 33.17 | | (±18.54) | | 31.80 | | | (±14.80) | | 0.871 |
| SUV = standardized uptake value, SUL = standardized uptake value normalized by lean body mass, TFL = contralateral lung compared to the side of the tumor. | | | | | | | | | | |
